# Functional Synchronization: The Emergence of Coordinated Activity in Human Systems

**DOI:** 10.3389/fpsyg.2017.00945

**Published:** 2017-06-13

**Authors:** Andrzej Nowak, Robin R. Vallacher, Michal Zochowski, Agnieszka Rychwalska

**Affiliations:** ^1^Department of Psychology, SWPS University of Social Sciences and HumanitiesWarsaw, Poland; ^2^Department of Psychology, Florida Atlantic University, Boca RatonFL, United States; ^3^Department of Physics and Biophysics Program, University of Michigan, Ann ArborMI, United States; ^4^The Robert Zajonc Institute for Social Studies, University of WarsawWarsaw, Poland

**Keywords:** synchronization, function, self-organization, mind, brain, social systems

## Abstract

The topical landscape of psychology is highly compartmentalized, with distinct phenomena explained and investigated with recourse to theories and methods that have little in common. Our aim in this article is to identify a basic set of principles that underlie otherwise diverse aspects of human experience at all levels of psychological reality, from neural processes to group dynamics. The core idea is that neural, behavioral, mental, and social structures emerge through the synchronization of lower-level elements (e.g., neurons, muscle movements, thoughts and feelings, individuals) into a *functional unit*—a coherent structure that functions to accomplish tasks. The coherence provided by the formation of functional units may be transient, persisting only as long as necessary to perform the task at hand. This creates the potential for the repeated assembly and disassembly of functional units in accordance with changing task demands. This perspective is rooted in principles of complexity science and non-linear dynamical systems and is supported by recent discoveries in neuroscience and recent models in cognitive and social psychology. We offer guidelines for investigating the emergence of functional units in different domains, thereby honoring the topical differentiation of psychology while providing an integrative foundation for the field.

## Introduction

Humans perform an astonishing array of activities with varying degrees of complexity, and they do so at a wide range of operational levels. On even the most mundane day, people prepare and consume meals, engage in physical exercise, plan activities, socialize with acquaintances and friends, drive a car and navigate traffic patterns, compose messages and letters, play games, accommodate their behavior to meet the demands of informal and formal social situations, daydream, and think about their personal qualities and weaknesses. On less mundane days, they may create music, write an essay or compose a poem, develop a theory, attempt to resolve a conflict, coordinate with other people to accomplish complex tasks, or play *Pokémon Go*. Each of these activities represents operations involving brain function, movement, perception, and higher-order cognition, and many of them also involve social interaction and coordination with other people who have their own personal and interpersonal agendas.

These activities and levels of operation are typically investigated in terms of their local dynamics, an established approach to understanding that has given rise to the highly compartmentalized discipline of psychology. Neuroscience, judgment and decision-making, and group dynamics, for example, tackle very different facets of human experience, and do so with little attention to possible underlying principles that provide integration for them. With this lack of theoretical integration in mind, our aim in this article is to suggest that the different activities and operational levels characterizing human experience can be understood in terms of a common process that has potential for forging a unified account of psychological functioning.

The core idea is that all operational levels of human activity, from brain function to group dynamics, represent the formation of *functional units* that result from the tendency for lower-level elements to achieve coordination and operate in concert to accomplish tasks. More specifically, we propose that functions in neural, psychological, and social structures emerge by the dynamic creation of functional units that are established by assembling a set of synchronizing lower-level elements into a coherent structure. This hypothesis has its well spring in principles of complexity science and non-linear dynamical systems and receives tentative support from recent discoveries in neurophysiology and recently developed models in psychological and social science.

## Processes of Synchronization

Brains, motor behaviors, minds, dyads, and social groups are clearly very different from one another. Brains are composed of neurons, motor behavior involves muscle contractions and limb movements, human minds represent the expression of thoughts, perceptions, and emotions, dyads consist of interacting individuals, and groups consist of many individuals in interaction. The elements in each case—neurons, muscle movements, thoughts and feelings, individuals—are clearly distinct by almost any criterion. From another perspective, however, these phenomena share important features. Each represents a complex system composed of many lower-level elements, and the operation of each system involves mutual influences among these elements.

We propose that these similarities across levels can be conceptualized in terms of common mechanisms by which any complex system performs a function. In broad terms, cooperative activity among elements is the essence of effective performance in any system. In more precise terms, the performance of a function requires the synchronization of specific elements, and changes in the configuration of these elements as the function unfolds in response to task demands.

### The Meaning of Synchronization

Synchronization can be described from two perspectives: at the level of system dynamics and at the level of influence among system elements. At the system level, synchronization refers to the coordination in time among the states or dynamics of the elements comprising the system (e.g., Schmidt and Richardson, 2008). With respect to the brain, this aspect of synchronization is manifest as in-phase relations in the activation of neural elements, or locking to an externally driven oscillatory signal (Buzsaki, 2006), although more complex forms of coordination are possible and have been observed. With respect to motoric behavior, the contraction of different muscle groups must be coordinated in time to coalesce into an activity (e.g., Bernstein, 1967; Turvey, 1990; Thelen, 1995; Kelso, 1997). With respect to the mind, an ensemble of cognitive and affective elements must be mutually consistent to generate a higher-order mental state such as an attitude, belief, or value (e.g., Thagard and Nerb, 2002). With respect to dyadic interaction, the overt behavior and internal states (e.g., emotions, attitudes) of the individuals must achieve coordination in time in order for the interaction to proceed smoothly (e.g., (Newtson, 1994; Fusaroli et al., 2014). And with respect to social groups, collective performance of any task requires the coordination in time of individuals’ activities (e.g., Arrow et al., 2000).

At the level of elements, synchronization can be viewed in terms of mutual influence, with consistent signals arriving at an element from other elements (Singer, 1999; Engel and Singer, 2001; Uhlhaas et al., 2009, and references therein). In the simplest attractor neural networks, for example, correct recognition of an incoming pattern is associated with each neuron receiving relatively congruent signals regarding its state from all the neurons with which it has connections (Zochowski et al., 1993). With respect to motoric behavior, each muscle relevant to the behavior must receive congruent signals from the other relevant muscles in order to perform the behavior (e.g., Bernstein, 1967). With respect to the mind, a coherent view or attitude is experienced when the thoughts that arise in consciousness call to mind other thoughts that support the same view or attitude (e.g., Abelson et al., 1968; Tesser, 1978). In dyads, the separate components of each person’s behavior (e.g., posture, facial gestures, postural cues, tone of voice, and speech content) coalesce into a coherent message (e.g., expressing an internal state, conveying an expectation, etc.) (e.g., Fusaroli et al., 2014). With respect to social groups, effective collective action depends on each group member receiving clear signals from other group members regarding of his or her contribution to the group effort (e.g., Forsyth, 1990). For example, attempting to synchronize one’s walking with others marching in a parade is an easy task when the others are synchronized because the signals from them concerning one’s suggested movements are consistent. If, however, the group is not synchronized, the signals arriving from different individuals are conflicting.

Both perspectives on synchronization—the temporal coordination of dynamics and congruence in signaling among elements—represent the binding of dynamics (i.e., the dynamics of one element is dependent on the dynamics of another element). Such binding does not necessarily involve performing the same action at the same time, but rather may involve compensatory dynamics. A group, for example, can have complex forms of synchronization if there are different tasks to be performed. This is clear in a band, for example, where each member plays a different instrument, yet each instrument informs the other instruments where it is in the musical piece and what sound should be made at each moment.

The basic hypothesis that synchronization plays a crucial role in the emergence of functions, both within and between levels, is consistent with several lines of research from complex systems, social and cognitive psychology, and social science. The present model, however, extends existing models by identifying mechanisms by which the synchronization of elements occurs. In particular, it identifies a dynamic scenario in which synchronization is an intermittent phenomenon characterized by the repeated assembly and disassembly of elements in accordance with shifting tasks and challenges faced by the system.

### Assembly of Functional Units

Functional units may be mobilized in three ways that reflect the emergence of synchronization. First, synchronization may result from the structural connections among system elements; some of the elements of the system may be connected to other elements in a manner that is more or less stable, which creates the potential for communication and therefore mutual influence. Mutual influences through these links can establish synchronization, even if the links are relatively weak (Pikovsky et al., 2003, and references therein; Strogatz, 2004). Activation of each of the elements sends signals to other connected elements, resulting in synchronization among the elements of the whole assembly. Each instance in which a functional unit is assembled strengthens the connections between the elements, paving the way for the next appearance of the same configuration. In effect, if functional units arise on the basis of structural connections, they tend to recreate the same configuration of elements in consecutive emergence of the units.

This assembly process can be observed at the level of the brain, the mind, and social groups. In the brain, neural structures that possess anatomically systematic and direct connections with each other will tend to synchronize. Such connections facilitate synchronization either by transmitting excitatory and inhibitory impulses (Buzsáki and Draguhn, 2004; Buzsaki, 2006) or by modulating intrinsic neuronal properties of connected neurons (Bogaard et al., 2009; Fink et al., 2012, 2013; Knudstrup et al., 2016). In mental systems, the co-occurrence of cognitive elements creates new associative links and strengthens existing links between elements. At the social level, repeated synchronization between individuals increases their liking for one another and strengthens their interpersonal relations. Family and friendship ties, for example, can serve to synchronize the thoughts and actions of the individuals involved. In similar fashion, close friends are likely to cooperate in the achievement of diverse goals.

If the interactions between elements are reflected in structural connections, the stability of these connections will facilitate the recreation of similar (or identical) assemblies of elements. If a highly trained mechanism is disrupted, it is easy to re-establish. This is easy to appreciate in stable social groups. If the members of a family take a vacation in different places, for example, they are likely to reunite once their vacations have ended. However, if the elements are connected by quick-changing bindings of dynamics, a momentary alteration of the functioning of relations between the elements may contribute to an emergence of distinct functional units. Even a small disturbance of a newly formed mechanism may cause qualitative changes in its performance. Therefore, if someone or something divides a group of persons who randomly had a conversation on a street, they may never reunite again.

Second, elements are likely to achieve mutual synchronization if they become salient in some manner at the same time. This mechanism is likely to be used to synchronize elements that are instrumental to the achievement of a goal. Activation of these elements by an internal control process (e.g., attention) can result in their emergent synchronization. On the level of the brain, attention can momentarily bind the dynamics of elements (Lopes da Silva, 1991). As an example of this mechanism, Wróbel (2014) hypothesized that during perception, attention is mediated through activation of selected neural groups though oscillations in the beta band, that in turn are being synchronized to form specific representations, in the gamma band (Wróbel, 2014). On the level of mind, recalling elements that are relevant to a judgment or a decision activates these elements, which are then likely to be synchronized into a judgment or become the basis for a decision. In social groups, individuals who have skills instrumental to solving a group problem or the achievement of a goal are often explicitly or implicitly called upon, promoting the formation of a team that synchronizes to perform the function.

External factors may also induce momentary synchronization among a set of elements by selectively activating them. At the level of the brain, sensory input can activate distinct neural assemblies in the brain, with this heightened activation creating the potential for mutual influence among the respective assemblies. Impression formation exemplifies this mechanism at the level of the mind. Thus, those features that distinguish a person in a given context will be integrated into the resultant impression, while other features are likely to be neglected (e.g., Asch, 1946). At the social level, meanwhile, if a few people stand out as the most active and expressive in a large group, they are likely to become coordinated in some fashion because the activity of each is most visible to the others. Therefore, the persons who are active in a given situation begin to act spontaneously and have greater chances of creating a functional unit—in this case, a subgroup performing a task. There is a positive feedback loop between momentary synchronization and momentary influence among elements, such that coherent elements influence one another more strongly and elements that influence one another become increasingly synchronized (Waddell and Żochowski, 2006).

In the third mechanism, the state or the actions of each element suggests the possible range of states and actions of other elements. In neural networks, this phenomenon is described as *multiple constraints* and it is one of the basic mechanisms by which artificial neural networks function (McClelland and Rumelhart, 1986). This can be observed at the level of dyads and social groups; it is described as social codependency in game theory and as affordance categories in the ecological approach (Gibson, 2014). An example of codependency is a situation in which an individual stepping right or left makes this position unattainable for the other person. An analysis of reciprocal delimiting of one’s own affordances is an important mechanism in the dynamic analysis of codependency in sport. For example, synchronization of soccer players is partially a result of the fact that players of one team block their opponents in order prevent them from performing certain actions, thus reducing their affordance (Vilar et al., 2013). Synchronization of elements can thus emerge not only as the result of some elements inducing others to be in a specific state, but also by elements dynamically limiting the ensemble of states that the other elements can adopt. This mechanism can provide for complex patterns of synchronization in functional units.

### Dynamics of Functional Units

Most models emphasizing the emergence of functions through synchronization of lower-level elements typically assume a static framework, in which the dynamics (if any) are limited to simple externally or internally imposed tasks. Dynamic processes play a more prominent role in the present model, promoting sustained change in the structure and functioning of the system in question. The core idea is that in carrying out higher-order functions, various configurations of elements are composed and decomposed along with the development and achievement of the function. Once a function is accomplished, the set of elements may be disassembled, ready to be reassembled in a different manner to perform a different function. New functional units may also be subject to decomposition by a control mechanism; this takes place when the elements are unable to achieve sufficient coherence necessary for the unit to carry out its functions. The present model, in other words, emphasizes the *intermittent nature* of synchronization, with the repeated assembly and disassembly of functional units in response to changing tasks, challenges, and environmental constraints. Thus, synchronization is not a mere consequence of functioning but also an important component of self-regulatory control (Żochowski and Liebovitch, 1997, 1999; Żochowski and Dzakpasu, 2004; Waddell and Żochowski, 2006).

The dynamics underlying the assembly and disassembly of functional units mirror one another. Whereas increasing synchronization strengthens momentary influence among elements and thus creates a functional unit, decreasing synchronization weakens the momentary influence among elements and thus disintegrates the functional unit. Regardless of whether the initial factor is weakening of momentary influence or breakdown of synchronization, the functional unit disintegrates. These elements then may become integrated into different functional units.

Whether the system will organize the same elements into the same functional units depends on the degree to which the emergence of the functional unit is dictated by the structural properties (i.e., couplings between elements) as opposed to the temporary binding of dynamics induced by momentary synchronization. If the elements influence one another primarily by structural linkages, the relative stability of the connections will result in the re-emergence of similar, if not identical ensembles of elements. A highly automatic or overlearned response, for example, may be temporarily disrupted but is easily re-established in the same form. In like manner, synchronizing neural groups form different spatial patterns in different tasks, reassembling their coordination whenever the function performed requires it (e.g., Kelso and DeGuzman, 1991). If, however, the elements are coupled primarily by fast-changing bindings of dynamics, momentary changes in the functional relations between elements can make the re-emergence of the original configuration unlikely, promoting instead a vastly different functional unit. In performing a relatively novel act, for instance, even a slight disruption can promote a wholesale change in the action (Vallacher and Wegner, 1987).

Function imposes constraints on synchronization. Even the same act might involve different configurations of lower-level elements in order to perform a particular function. When hitting a chisel with a hammer, for example, professional blacksmiths unconsciously coordinate arm muscles to maintain precision from strike to strike. However, such precision is not present on the level of a single muscle. In one strike, a particular muscle might be more engaged than in another strike, with another muscle compensating for the muscle’s lack of engagement (Bernstein, 1967).

## Synchronization in Psychological Processes

The functional role of synchronization can be seen at all levels of psychological reality: brain function, perception, motor behavior, higher-order action, mental processes, dyadic behavior, and collective action in social groups.

### Stimulus Representation and Consciousness

Synchronization plays a crucial role in how the brain performs its functions. Brain function requires both the segregation and integration of information, whether sensory or retrieved from memory. With the development of techniques for visualizing brain activity, we know relatively well how the brain segregates such information by specifying distinct regions for processing specific types of information. Our knowledge about how the brain integrates information, however, is much more limited. The leading hypothesis relates information integration to synchronization between regions processing different types of information (e.g., von der Malsburg, 1994; Singer and Gray, 1995). Synchronized activity of neural assemblies in the brain is theorized to be important to the performance of sensory and perceptual functions (von der Malsburg, 1994). Synchronized oscillations between brain regions have been observed in motor and cognitive functions, specifically in conscious processing (von der Malsburg, 1994; Tononi et al., 1998). Sensation of the simplest object requires the synchronized activity of neural ensembles (cf. Tononi and Edelman, 1998; Sauvé, 1999; Engel and Singer, 2001). Moreover, long-range synchrony between distant brain regions is observed in multiple forms of behavior (Harris and Gordon, 2015). Correlation code is also thought to underlie selective attention (Niebur et al., 2002; Gomez-Ramirez et al., 2016).

To understand how synchronization of neural activity could fulfill the role of information integration, we need to realize what a daunting task it is to combine inputs from so many dispersed and functionally distinct sources. The binding problem represents the prototypical challenge for integration of information in the brain. If a person is perceiving a blue circle and a red square for example, how does the brain bind the shape and color features to form a representation of the object? In other words, how does the brain know that the circle is blue and the square is red?

Singer and Gray (1995) proposed that temporal characteristics of the neural activity are responsible for the binding, such that all the neuronal groups coding different features of the same object will synchronize their activity to within the range of milliseconds. This process enables integration of multiple features and the concurrent performance of multiple perceptual functions, such as the integration of features into several distinct objects. This can be achieved by using distinct temporal patterns (e.g., frequency and phase differences) for the performance of each function (i.e., integration of each object’s features). The same mechanism may explain hierarchical organization, where one group of neurons belongs to more than one integrative unit at the same time (e.g., through synchronization on harmonic frequencies). The temporal correlation hypothesis also explains how integrated wholes may interact at higher levels of information processing, as synchronized neural assemblies form a functional unit at a higher level, which is distinguishable from other neural assemblies because of its particular temporal pattern. Synchronized neural assemblies are more visible than are unsynchronized assemblies, even if the former are smaller, because a neuron is much more likely to produce an action potential if the incoming signals from its input neurons are synchronized.

Such binding must occur across virtually all modalities: auditory binding may be needed to discriminate the sound of a single voice in the crowd, and binding across time is required to perceive the motion of the object. A cross-modal binding is required to associate the sound of a ball striking a bat with the visual percept of it, so both can be perceived as different aspects of the same event. Cognitive binding, for example, must link visual perception of an object with its semantic knowledge, memory reconstruction, and cross modal identification (see *Neuron, 24*, 1999, for a review). Synchronized activity is mostly visible (and recorded) as synchronous oscillations in the electrical activity between various brain regions. Interestingly, gamma-band synchronous oscillations (GSO) of neural-electrical activity are believed to bind sensory sensations to represent distinct objects (Buzsaki, 2006; Buzsaki and Wang, 2012) and attention is mediated through activation of selected neural groups though oscillations in the beta band (Wróbel, 2014).

At each level of information processing, synchronized groups form functional units that integrate into increasingly complex structures. These neuronal groups from different brain regions may correspond, for example, to personal memories, affective reactions, and so forth, with respect to the object. Each assembly at a lower level may be responsible for detecting specific features of the stimulus, but it is the synchronized representation of the various assemblies that gives rise to conscious awareness of the object. Such a synchronized neural group is similar to the notion of *cell assembly*, as proposed by Hebb (1949), in which intragroup connections facilitate activation of the entire group when a single neuron is activated. This, in turn, strengthens the within-group connections, as epitomized by the phrase, “cells that fire together wire together.” In effect, the strength of coordination partially depends on the history of learning and is represented by changes in the strength of synaptic connections (i.e., changes that occur on a relatively slow time-scale) that accompany learning.

The temporal correlation hypothesis does not require the formation of stable structural connections, but rather proposes that temporal strengthening of synapses (LTP—long-term potentiation) may also be responsible for the creation of a synchronized functional unit. Functional units are therefore dynamical formations appearing for a short time and disassembling shortly thereafter, allowing for the creation of new functional units (Rychwalska, 2013).

To a certain extent, the interaction among elements may also change on an even more intermittent basis due to changes in focus of attention (e.g., Friston, 1994; Maunsell, 1995). Attention, in other words, brings together diverse groups of neurons that then have the opportunity to synchronize with one another.

The functional unit highest in the hierarchy that can be described in the brain activity is possibly a unified conscious “scene” (Tononi and Edelman, 1998)—a representation of a time frame in the stream of consciousness. Such high integration requires long-range correlations and complex temporal patterns of coordination. In other words, functional binding between distinct neural assembles has to be highly flexible, enabling the functional cluster to move through a sequence of distinct states without losing its synchronization (Koch et al., 2016; Palva, 2016; Ward, 2016; cf. Nakatani et al., 2013). At the same time, loss of consciousness itself (e.g., due to anesthesia) is generally associated with “cognitive unbinding” (Mashour, 2013 and references therein) and is thought to be mediated by loss of long range synchrony in the brain (Lewis et al., 2012).

### Higher-Order Mental Process and Structure

Once conscious representations are formed (in accordance with the scenario outlined above), they become elements subject to further integration processes that result in higher order mental structures such as action representations, judgments, and self-concepts. As with the brain, synchronization plays a crucial role in this process. If the process of progressive integration can maintain synchronization among a subset of elements, it proceeds until a cognitive function is performed (e.g., a judgment, a meaningful action, a new insight into self), which in turn is subject to further integration processes, and so on.

Considerable research has established that coherence is indeed a basic principle in cognitive function and structure (cf. Abelson et al., 1968). Within this framework, a variety of mechanisms have been identified whose function is to maintain coherence in the face of incongruent information or social influence (e.g., dissonance reduction, discounting, selective memory, etc.) (cf. Tesser et al., 1996; Swann, 1997).

The nature of the cognitive function dictates the specific metric by which coherence is assessed. In forming a judgment of someone, the function is the establishment of an unequivocal behavior orientation toward the person (cf. Jones and Gerard, 1967). In self-understanding, the function is self-assessment (cf. Tesser and Campbell, 1983). In action representation, the function is effective performance (cf. Vallacher and Wegner, 1987). In each case, the issue of coherence is how well the elements support each other (i.e., coordinate) in achieving their respective function. Thus, a coherent social judgment is one in which all the activated cognitive elements are consistent in their implications for evaluation of the target. In self-understanding, meanwhile, a coherent self-concept is one in which activated self-relevant information paints the same evaluative portrait. And in action, a representation is effective to the extent that the lower-level action features synchronize to produce a fluid performance (cf. Vallacher et al., 1989; Csikszentmihalyi, 1990).

When coherence among elements cannot be achieved in the process of progressive integration, control mechanisms disassemble the emerging structure and attempt to coordinate the elements or a new set of elements. This process may be repeated until the function is achieved (i.e., a coherent judgment is reached or an effective action is performed) or, alternatively, it is possible for the disassembled elements to become reconfigured into an entirely different functional unit. A new function, in other words, may emerge from the disassembly and subsequent reconfiguration of cognitive elements (Vallacher et al., 1998). In action, for example, an inability to maintain the act of “persuading someone” may lead to a reconfiguration of one’s speech acts as “expressing oneself.”

The functioning of mind may thus be described as the continual assembly and disassembly of cognitive elements in the search for coherence. The stream of consciousness may ultimately be a tumbling ground for whimsies (James, 1890), but this very feature of thought enables the emergence of structure and effective function. The progressive assembly and disassembly of system elements is reflected in the temporal trajectory of emergent thought. In social judgment, for example, univalent (evaluatively congruent) information is organized into progressively higher level structures reflecting increased coherence, a scenario that is reflected in thought-induced attitude polarization (Tesser, 1978). Mixed valence information, however, tends to result in the repeated assembly and disassembly of differently valenced elements in a process of dynamic integration (cf. Vallacher et al., 1994; Vallacher and Nowak, 1997). The process of progressive integration has also been observed with respect to self-reflection, with individuals who are instructed to focus on the details of their action displaying increasing oscillations in their self-evaluations during self-reflective thought, indicative of the assembly of progressively higher-order evaluatively coherent structures (Vallacher and Nowak, 1999; Vallacher et al., 2002).

From the perspective of synchronization, coherence of cognitive representations is fundamental. Coherent representations will be integrated into higher-order representations, while incoherent ones will either be disintegrated or will have their incoherent parts eliminated in the process of integration. From this standpoint, the signals of coherence are global cross-modal signals. Coherence in one sensory modality favors progressive information integration in other modalities; incoherence in one modality disrupts signal integration taking place in different modality. Research has shown that watching incoherent figures evokes a sensation that a musical selection does not follow familiar principles, while watching coherent figures facilitates the feeling that such music is familiar (Ziembowicz et al., 2013; Winkielman et al., 2015).

Despite the deep roots of this perspective in classic treatments of mind (e.g., James, 1890; Kohler, 1929; Wertheimer and Riezler, 1944; Asch, 1946), the traditional approaches to modeling cognitive function have typically portrayed the mind as a stable organization of knowledge. Connectionism has emerged in recent years as the tool of choice in investigating how systems resolve conflict and maximize coherence (cf. Read and Miller, 1998). Thus, the function of cognitive networks is assumed to be the satisfaction of multiple constraints (represented by connections), such that the network achieves a configuration in which the states of nodes are least conflictful. Although connectionist models can solve the coherence problem, they have an important limitation with respect to modeling the scenario we have described. In particular, most models are limited to a single step, in that once a coherent solution has been achieved, the system is trapped in this state and does not evolve further.

### Action Control

Minds do not exist for their own sake, leaving people “buried in thought” (Tolman, 1951). The mental content and structures that emerge in line with the synchronization scenario outlined above provide the basis for overt behavior in the context of environmental constraints, challenges, concerns, and personal goals. Because the local environment for action is subject to noteworthy and continual changes, people’s mental representations must be dynamic as well, undergoing reconfiguration when necessary to promote and maintain effective action and to repair ineffective action. This scenario of repeated assembly and disassembly of mental representations in service of effective action is central to action identification theory (Vallacher and Wegner, 1987). The theory holds that effective performance of an action is associated with progressive integration of the lower-level structural elements of the action. This integration of elements into a higher-level functional unit promotes a corresponding shift in the person’s mental representation of what he or she is doing. A novice tennis player, for example, is likely to identify his or her behavior in terms of the basic acts involved—adjusting body position, swinging the racket, and so forth. As these basic acts become sufficiently synchronized to promote effective play on the tennis court, the person’s identification of the action will change accordingly to a more integrative (higher-level) representation—“playing tennis,” “getting exercise,” or perhaps “competing against an opponent.”

By the same token, if the action becomes ineffective when identified at a particular level of identification, the person is likely to shift to a lower-level identification that reflects the basic structural elements of the action. The tennis player who fails to play tennis effectively, for example, may regain mental control of the action by refocusing his or her conscious attention on shifting his or her body position and swinging the racket. Through this scenario of repeated assembly and disassembly of mental representations of action, people eventually converge on an optimal level of action identification that reflects the degree to which the action’s structural elements are synchronized and constitute an effective functional unit (e.g., Vallacher et al., 1989).

The emphasis on the cognitive representation of action in this scenario may seem at odds with a large body of research on behavioral coordination (e.g., Bernstein, 1967; Kelso and DeGuzman, 1991; van Wijk et al., 2012). Researchers in this area have emphasized that reactions to changing environmental circumstances and skill acquisition do not require conscious mental representations. Instead, there is a direct coupling of perception and action, such that environmental affordances are registered at a perceptual level without the need for higher-level cognitive interpretation. Environmental affordances also shape motor reactions through coupling of behavior and perception, such that refined and skillful enactment of behavior leads to finer distinctions in the perception of the context in which the action unfolds.

This perspective holds that in developing a motor skill, the specific movements become coupled, so that the system as a whole loses degrees of freedom (e.g., Bernstein, 1967; Turvey, 1990). So although hundreds of muscles are involved in even such an act as shaking hands, for example, it is unlikely that the central nervous system could cognitively cope with the control of each muscle. Bernstein (1967) suggested, however, that muscles form function-specific synergies—self-organizing assemblies—by locally coupling and constraining each other’s contractions. These patterns of mutual constraint are flexible, changing in accordance with the requirements of the function. The patterns of coordination among hand muscles, for example, is different when hitting than when grasping. The patterns of coordination are also context-specific. So even when performing the same task, the pattern of coordination may be quite different. Operating a wrench may require different muscle configurations when it occurs in a confined space (e.g., under the hood of a car) than when it occurs in an open space (e.g., on a workbench).

From the perspective of action identification theory, skills acquired at the motor level (e.g., the coordination of inter-limb movement configurations) correspond to the lowest levels of action identification. As the action becomes progressively mastered or habitual, patterns of motor coordination become non-conscious elements in higher-order units that are increasingly accessible to conscious representation. Once conscious representation of an action’s higher-level meaning is achieved, however, the lower-level automated elements can, in principle, become subject to conscious representation as well. Learning to walk, for example, occurs without thinking about how to move one’s legs; rather, it involves trial and error in service of navigating the physical environment. Although walking remains largely automatic once it is learned, such that its elements (e.g., shifting weight) are not mentally represented, circumstances may arise that bring these elements into consciousness. Thus, a slippery floor might focus a person’s conscious attention of how he or she is shifting his or her weight and moving his or her legs. So although the mutual constraints promoting patterns of movement coordination may develop without conscious control, they may subsequently become subject to conscious control and modification.

### Dyads

In dyads, any interaction (e.g., conversing) or task (e.g., problem solving or moving a box) requires synchronization at various levels, including motoric behavior and internal states (emotions, thoughts) (e.g., Nowak et al., 2000). The development of interpersonal synchronization is well documented. In conversations, for example, individuals spontaneously synchronize their facial expressions (e.g., Stel and Vonk, 2010). This effect is so prevalent that people will even mimic the facial expressions of an inanimate object—for example, a robot (Hofree et al., 2014). Synchronization of facial expressions, in turn, tends to promote the corresponding emotional state in each member of the dyad, in line with the facial feedback hypothesis (e.g., Laird, 1974; Strack et al., 1988).

Computer simulations of dyadic interaction have shown the relationship between synchronization patterns and the inner properties of the two coupled units (individuals) takes diverse, and often quite unexpected, forms (Nowak et al., 2002). Although small changes in the dynamical properties of either unit may promote correspondingly small differences in synchronization, sometimes even very minor changes in these properties will produce qualitative changes that can be interpreted as phase transitions in the form of coordination.

When we take into account the complex dynamics associated with each individual, the higher-order system created by two individuals can become capable of especially rich dynamic properties, generating rich and complex patterns of coordination. The observed forms of coordination go beyond simple in-phase synchronization and anti-phase synchronization to include considerably more complex forms (Nowak et al., 2005). The complexity of two coupled systems may greatly exceed the complexity of each of the component systems (i.e., individuals)—or it may become drastically simplified in a scenario resembling the control of chaos (Ott et al., 1990).

Conversation is an especially important form of dyadic interaction. Fusaroli et al. (2014) argue that function is critical in organizing interpersonal synergy in a dialog. Beyond simple in-phase synchronization, the individuals in a dialog display complementary dynamics, with one person compensating for the other with respect to mistakes and perturbations. The two individuals become integrated into a higher-order unit that, in turn, influences their respective cognitive, linguistic, and motor processes aimed at achieving a common goal. Synchronization, in other words, occurs at multiple levels, both within and between the individuals.

The pattern of synchronization is modulated by the function of the interaction and by the interaction context. Thus, the mode of synchronization that is functional in one context might be dysfunctional in another context. For example, repeating simple utterances of a partner might be functional in a highly structured situation (e.g., repeating commands to ensure accuracy of communication), but would be awkward and redundant—hence, dysfunctional—in a unstructured social conversation.

Interactions in a dialog serve to distribute cognitive processes and actions between the individuals following the demands of the task and each individual’s capacities. The dyad, then, becomes a higher-order unit capable of achieving more than what can be achieved by the individuals behaving alone. The function is defined at the level of the emergent dyadic whole rather than at the level of each individual. Fusaroli et al. (2014) argue that this process of organizing interpersonal interactions in a dialog is structured in service of a joint function rather than in the separate cognitive systems of the individuals. The interaction patterns are characterized by stability and clear ordering of the dynamics of both individuals (e.g., the rhythm of a conversation). The functionality of dyadic dialog is clearly visible in *dimensional compression* (Bernstein, 1967). This means that the collective variability in joint coordinative tasks is less than the variability of each individual’s movements, in analogy to the coordination involved in an individual’s performance of a task, as described earlier (p. 10).

### Groups

A social group is not only a set of people, the relations between them, and the social structure, but also the continuous process of synchronization of gestures, looks, acts, and communication (cf. Arrow et al., 2000). The achievement of a group task depends upon such synchronization (cf. Forsyth, 1990; Schmidt and Richardson, 2008; Marsh et al., 2009). Decision-making requires the coordination of information and opinions, for example, while the performance of a group action requires the synchronization of the actions of the group members. Synchronization also establishes group structure. Social relations, in fact, may be defined in terms of categories of synchronization (Baron et al., 1994; Newtson, 1994; Nowak et al., 1998; Marsh et al., 2009; Miles et al., 2009). Synchronization with group other members leads to the formation of social ties and promotes a feeling of connectedness (e.g., Chartrand and Bargh, 1999; Lakin and Chartrand, 2003; Dijksterhuis, 2005), while the inability to achieve synchronization evokes feelings of solitude (Nowak and Vallacher, 2007).

In the pursuit of coordination, individually conditioned behaviors merge into regular patterns of joint action (Guastello and Guastello, 1998; Marsh et al., 2009). The emergence of coordinated behaviors may be operationalized as a correlation in time between the internal states and the behaviors of individual members of a group. A group is more predictable (i.e., it has fewer degrees of freedom) than any of the individuals considered separately. This means that the behavior of group members both limits and is limited by the behavior of other members. Although participants of a group discussion take the floor independently, for example, they do so in the context of what has already been said.

Different challenges and tasks may require different patterns of coordination. A task may require negative feedback (reciprocal dampening of reactions), enacted by criticism, for example, or by reducing the number of possible decision variants. Alternatively, the task may require positive feedback intended to generate many ideas, motivate one another to work, or otherwise contribute to the group effort. When a group focuses on making a final decision between two options, for example, a discussion may involve a sequence of statements alternately expressing arguments for each of the options. Also, an increased number of “we” messages may appear in participants’ references to the task at hand, since the group functions as a whole to make a collective decision or an action plan.

Momentary coordination of group members engaged in a discussion or a collaborative activity is a sinusoidal process—it rises and falls from moment to moment along with the work of the group. In a given moment of a group’s duration, the behavior of its members organizes itself around a task to be performed or an issue to be discussed. The members of the group commence collaboration in order to carry out a task or to convince others to agree with a particular opinion. Temporary increases of coordination may be described as an emergence of functional units serving the purpose of carrying out micro-tasks. A given pattern of coordination between the participants breaks down immediately after a given objective is reached or a thread of the discussion runs out.

It is not necessary for the entire group to be synchronized; rather, different subsets of individuals will synchronize to accomplish a task and then de-synchronize once the task is completed (e.g., Sawyer, 2005). Over time, then, a group can be characterized by the emergence and disassembly of different interaction patterns reflecting the synchronization of various subsets of group members. Ziembowicz (2015), for example, demonstrated that in task-oriented groups, the momentary emergence of dyadic interaction structures tended to characterize the appearance and resolution of interpersonal conflict. Interactions involving more than two individuals, however, tended to be associated with more positive affect, weaker opinions, and greater inquiry. Different emergent social structures, then, carry out different functions in social groups.

The coordination of group members’ behaviors occurs through their reactions to one another, and through the exchange of gestures, looks, and messages. But coordination can also occur on a deeper level with respect to emotions, judgments, beliefs, and action plans (cf. Nowak et al., 1998). Group-level synchronization is sometimes manifest as emotional contagion, for example, whether in face-to-face contact (e.g., Hatfield et al., 1993) or in social networks (e.g., Kramer et al., 2014). Research (Nowak et al., 2005; Johnson, 2006) has shown that synchronization on a behavioral level is fundamental for the possibility of deeper levels of synchronization. Visual synchronization is especially important for the emergence of mutual positive emotions and empathy.

Several mechanisms promoting positive synchronization in interpersonal relations and groups have been identified. Similarity in attitudes, for example, is a basic principle of interpersonal attraction (e.g., Byrne et al., 1986), promoting the development of social ties between two or more individuals. Computer simulations of social influence (Nowak et al., 1990) have demonstrated that locally defined influence principles (e.g., social impact, Latane, 1981) lead to the emergence of locally coherent clusters of like-minded individuals (e.g., those with similar opinions or beliefs). Computer simulations of social interdependence have also demonstrated the emergence of locally coherent structures, where coherence is defined as similarity in strategies of interpersonal relations (e.g., Hegselmann, 1998; Nowak and Vallacher, 1998, Chapter 7; Axelrod, 2006). Mechanisms have also been identified that preserve and enhance interpersonal and group coherence, such as the rejection of deviates and the emergence of group norms (e.g., Festinger, 1950; Clore and Gormly, 1974; Latane, 1981).

The social ties that result from deeper levels of synchronization provide for increased influence among group members, analogous to synaptic connections in the brain and to associations in the mental system. A variety of factors apart from social ties, however, affect coordination in a group. For example, physical proximity momentarily magnifies the effective influence among individuals. The momentary salience of particular individuals (e.g., by virtue of physical appearance or behavior) can also affect the temporary configuration of links between individuals, magnifying some and weakening others. Momentary coherence (e.g., a shared mood or activity) can also reconfigure the links between subsets of individuals. Such coherence might be induced, for example, by some external signal such as music or highly salient events. In work groups, meanwhile, different structures of communication among group members tend to be associated with the emergence of correspondingly distinct modes of task solution and problem-solving (Leavitt, 1951; Shaw, 1951; Guetzkow and Simon, 1955).

Even in the context of existing social relations, not all interpersonal or communication links are activated at the same time. A person clearly has stable links to his family, for example, but these are not active when he or she is in some other social setting (e.g., work). In combination with the factors that operate independently of social ties (proximity, etc.), this suggests that social groups, much like mental and neural structures, have an assembly and disassembly aspect to them, reconfiguring themselves continually in response to changing environmental demands and contingencies.

Coordination among group members is typically associated with effective collective action. Beyond promoting strong and enduring bonds (i.e., cohesiveness) in a group (Forsyth, 1990), coordination has been identified as a critical factor in optimizing performance in work groups (Steiner, 1972) and sports teams (Vilar et al., 2013). At the same time, though, research has traced certain forms of dysfunctional group dynamics to global synchronization among interacting individuals. In “groupthink,” for example, a heightened concern with group cohesion can stifle dissent and thereby short-circuit natural self-correction tendencies (e.g., critical feedback, desire for individuation) that might otherwise prevent ill-conceived group decisions and actions (Janis, 1982).

Although existing relationships among the individuals in a group can promote the emergence of a collective functional unit, group-level synchronization can emerge in the absence of social ties. The phenomenon of “deindividuation” (Zimbardo, 1969; Diener, 1980), for example, refers to the loss of individual identity and self-awareness in large, unstructured groups engaged in a common action. This phenomenal state tends to produce heightened coordination of moods, thoughts, and actions among all the individuals in the group, which can promote irrational and sometimes violent behavior. The most extreme manifestation of global group synchronization is panic, where each individual tries to perform the same action (e.g., leaving through a single door from a burning building) without adopting a more functional mode of coordination (e.g., turn-taking). In their model of collective action, Turner and Killian (1957) noted that in unstructured group situations that today are seen as breeding grounds for deindividuation, there is often the spontaneous emergence of a group norm that synchronizes and maintains the actions of the group as a whole.

In sum, the coordination of individuals’ actions in a group or collective context—whether productive as in problem solving or seemingly irrational as in groupthink or deindividuation—represents the emergence of functional units. In this scenario, individuals represent lower-level elements that become synchronized, either through their mutual influence or through their common response to an external signal (e.g., a leader, a perceived threat or an opportunity). Groups are certainly distinct from neural systems, conscious representations, individual actions, and dyadic interactions, but they conform to the same formal scenario we have described for these other basic levels of psychological functioning.

## Synchronization Between Levels of Psychological Reality

This model can be used to understand the emergence of higher-order functional units at progressively higher levels of integration, linking neural, psychological, and social processes in a larger dynamical system. The idea that similar dynamical principles operate at different levels—from neural to behavioral to social—and that these levels influence one another in both a bottom-up and top-down manner has been articulated by complex systems theorists (e.g., Kelso et al., 2013).

In the bottom-up mode, synchronization of elements creates a functional unit that can then function as an element in further synchronization. By this means, synchronization at the level of the brain underlies the creation of thoughts and feelings. The synchronization of thoughts and feelings in an individual can then promote the emergence of his or her judgments and action plans. Once judgments and action plans are created within an individual, these higher-order mental states can synchronize with the judgments and action plans of other individuals with whom the individual is interacting. In a different route, patterns of synchronization among neurons in the brain can induce corresponding patterns of synchronization between muscle movements (Kelso et al., 2013), so synchronization of neuronal groups in the brain can induce behavioral synchronization in a direct way, bypassing cognitive representation. In both cases personal synchronization serves as the platform for dyadic synchronization. In a continuation of this process, synchronized dyads can synchronize with each other to promote effective group performance. On a dance floor, for example, dyads consisting of well-synchronized dance partners can navigate the dance floor, coordinating with other dyads and avoid colliding with them.

In this process, there are two types of transition between the lower-level and higher-level units. First, the synchronized ensembles of elements become unified into a single functional unit. Individuals who synchronize on a task, for example, become a team, which can then become an element in a higher level of organization—the work group. In this form of synchronization, higher-order units can be decomposed into its component lower-order units. In the second form of transition, a pattern of synchronization among elements at one level which can be described by an order parameter (Haken, 1987) may become an element on the higher level. Roughly speaking, an order parameter is a global variable that describes patterns of dependency among the elements of a system. Organization of system elements, as described by an order parameter, becomes an element in a higher-level system. The same set of elements, in other words, may be synchronized in different ways to produce correspondingly different values of the resultant order parameter. For example, the specific pattern of synchronization among neurons gives rise to specific thoughts and feelings. So in contrast to the first type of transition, it is the type of synchronization rather than the particular subset of elements that gives rise to the higher-level unit.

It is also the case that synchronization at a higher-level can promote patterns of synchronization at a lower level. Social interaction, for example, induces thoughts and feelings in the individuals, which can in turn influence their expectancies and patterns of attention, which can then induce synchronization at the level of neuronal activity. Attention, for example, induces synchronization in the beta frequency, which sensitizes the appropriate set of neurons to synchronize more readily in the gamma wavelength in the process of perception (Wróbel, 2014).

The bottom-up and top-down processes interact with each other in reciprocal feedback fashion, which creates a synchronizing dynamical system that can promotes continual modification and adjustment within and between levels. Synchronizing elements on the lower level self-organize into wholes with emergent properties on the higher levels. These emergent wholes, in turn, influence patters of synchronization of lower level elements. Two individuals interacting in a dialog, for example, form a dyad with properties that cannot be reduced to minds of the interacting individuals. The dyad, as an emergent whole, influences individual’s movements, language, cognitions, and emotions, which in turn influences the properties of the dyad (Fusaroli et al., 2014).

## Measuring Synchrony

The model we have presented brings new understanding of how functions are performed by systems at different levels—from mind to social groups. But the model has another benefit as well: the idea that functional units are assembled and disassembled to follow the demands of the task points to a novel way of defining and measuring functions. We can analyze what particular configurations of coordinating elements—be they neurons, concepts, or individuals—are required to perform specific functions.

### Functional Connectivity

To analyze the composition of a functional unit, we treat each synchronized pair of elements as a functional link. For a given period of time adjusted for the system under scrutiny—i.e., milliseconds for neural activity, seconds for the coordination of memories, or minutes for group discussion—we can then combine such existing functional links into a network. In this depiction, functional units’ properties can be analyzed with the help of network analysis. For example, we can measure the density of the functional unit: if the density is high (i.e., there are many coordinating pairs), we can assume that either the task performed is complex or requires redundancy. If the density is low, we can hypothesize that either the task is simple or the performing system has well defined roles for its elements. Other network measures—such as diameter or path lengths—can be used in similar way to understand both the dynamic requirements of the task as well as the system’s efficiency in performing it.

To date, network analysis has been the primary method for analyzing the structure of various systems. Possible dynamics and functions are usually inferred from the properties of structure (Watts and Dodds, 2007; Baronchelli et al., 2013; Weng et al., 2013). However, in very many systems—from brain through the cognitive system up to whole societies—the same structure of connections permits the system to perform various, sometimes diametrically different functions. Therefore, structural network analysis is not enough to understand how function is performed. Network science only partially acknowledges the problem through analyzing the changing structure of networks (Capocci et al., 2006; Holme and Saramäki, 2012). What we propose is to complement standard network analysis with the analysis of functional links dynamically formed by elements coordinating through stable, structural connections.

The dynamical approach to network analysis has to some degree tackled this issue by proposing the paradigm of time-dependent or temporal networks (Holme and Saramäki, 2012). This approach has evolved from the observation that most of the network systems analyzed do not “exist” for most of the time. For example, the huge networks of phone contacts only form a connected component (i.e., a network) if they are aggregated over many units of time (hours, days, months). If one were to analyze a single minute, at best the network would consist of many pairs of connected nodes. What has so far been analyzed as a network is usually just a set of possible (latent) connections that effectively exist only for limited periods of time.

Temporal network analysis is suitable in all those cases where the dynamics of the process going on the network is on a similar time resolution as the formation of the structure of the network. All networks dependent on face-to-face contacts (epidemics, opinion dynamics, etc.) or tele-contacts (phone, social media, texting, etc.) will fall in this category. In those cases, while it is still valuable to understand the network of latent connections, such analysis should be complemented by analyzing the dynamics of connection change as it severely affects various network measures (i.e., path lengths, reciprocity of connections, connected components, etc.).

Temporal network analysis has discovered that the network evolution over time in telephone contacts displays an interesting regularity. Certain connection sequences reappear more frequently than they should by chance (Braha and Bar-Yam, 2009; Kovanen et al., 2011). Such temporal network patterns—dynamical motifs—in phone calls are thought to reflect dynamics of the most common social processes over the underlying, stable structure of social acquaintance links (e.g., scheduling and feedback confirmation of meetings in a triad: A->B->C->A).

Dynamical motifs are a first step at analyzing not only structure, but also dynamics of a system as a network, which could help understand how a certain social process can be inferred from the changing structure of a network. We push this idea much further—we propose that certain spatio-temporal patterns of coordinating elements can be extracted from interacting elements and analyzed with network measures to show how a (relatively stable) structure of a system gives raise to many different functions, on different temporal scales.

### Functional Connectivity in Neural Systems

So far, this type of analysis has been used to study neural systems. There, it is especially easy to differentiate between structure and dynamics. Structural links are the (relatively) stable anatomical connections and functional links are the temporal dependencies between the activities of different neural regions (Friston, 1994; Baronchelli et al., 2013). Such links can be extracted at different temporal and spatial scales: from matching spike trains of single neurons or small neural assemblies, from correlated local field potentials of cortical columns as well as from phase locked EEG/MEG recordings from large cortical areas. Although the structural connections limit the possible functional connections, the relation is not unidirectional. Neurons that fire together, wire together (Hebb, 1949). That is, structural links are formed to strengthen the coordination patterns resulting from concurrent activation (i.e., common stimuli), sometimes distinguishing between millisecond differences in synchrony (Bi and Poo, 2001; Caporale and Dan, 2008).

Network analysis of *functional connectivity* has been successfully applied to brain function (Salvador et al., 2005; Stam and Reijneveld, 2007; Rychwalska, 2013). It has proven to be a useful methodology for the understanding and diagnosis of particular pathologies of brain function, such as Alzheimer’s disease (Stam et al., 2007), epilepsy (Ponten et al., 2007), and aging (Meunier et al., 2009). What is particularly promising is that analysis of functional connectivity reflects and differentiates between specific tasks—for example, singing from counting (Shirer et al., 2012) or passive observation from classification tasks (Krienen et al., 2014).

In this area, it is also clear how this method of analysis can also be used to measure the dynamics of functional unit assembly and disassembly. By depicting it as a dynamic network of functional links, we can analyze the change of network measures in time and understand how the demands of the tasks evolve. Functional connectivity networks in the brain change over time (Valencia et al., 2008), which suggests that they indeed evolve with the needs of the task.

### Future Directions

Although functional connectivity analysis has not yet been applied to mental processes or group functioning, it could prove to be a promising direction. In connectionist models of activation spread over memory or semantic network, for example, synchronization of activation of concepts can be easily portrayed as a functional network. The synchronous activation of various elements of the self-concept can also form a graph, with congruencies depicted as positive links and incongruencies as negative links.

In the analysis of groups, social network analysis is a rapidly developing research approach. However, it rarely recognized that configurations are meaningful for function of the social system (Johnson, 2013) or that links can be formed not only through structural connections (e.g., *Facebook* friends, contacts list on the phone), but also through functional ones (coordinated activity). Applying functional connectivity analysis to group function could illuminate how collective tasks present constraints on the required coordination patterns and how these patterns evolve to enable the group to flexibly switch between different functions.

The challenge for future research in this paradigm is to define meaningful markers of coordination in the respective areas (e.g., cognition, social interaction) that could be used to extract functional links with meaningful temporal resolution (i.e., allowing dynamical assembly and disassembly of functional units). Concurrent activation of certain concepts in the semantic network could be measured by combining physiological (e.g., eye-tracking) methodologies with computer-based methods (e.g., mouse tracking). In the social domain, the vast amounts of data traces collected by social networking through new media (the so-called Big Data) that often contain timestamps of activity could provide a valuable source of possible markers of coordinated activity.

## Conclusion

The model we have described offers a way to reframe distinct phenomena in terms of basic principles of synchronization dynamics. Whether the focus in the brain, cognition, social judgment, action, or group behavior, effective functioning is achieved through the synchronization of the lower-level elements at issue (neurons, thoughts, movements, opinions) to form functional units relevant to the task at hand. The coherence provided by the formation of functional units is often temporary, in place only as long as is necessary to perform the task. With changing task demands, then, there is repeated assembly and disassembly of different functional units, each providing the coordination necessary to perform a particular task demand. In this view, neural, mental, action, and social processes do not represent the output of static structures, but rather represent inherently dynamic systems that operate in accordance with a press for coherent functioning.

Although the importance of coherence in psychological systems is widely acknowledged across disciplines, the mechanisms by which coherence is achieved and maintained is not well understood, nor has there been an attempt to identify such mechanisms that are scalable across different levels of psychological functioning. The model we have presented is an attempt to provide this integration. Although there is tantalizing evidence in favor of this integration, the model is in its nascent stage and thus should be viewed as a heuristic for research agendas. With the appropriate degree of coordination of such research efforts, a comprehensive theory of psychological processes may emerge that can establish a functional scientific paradigm for the understanding of human experience.

## Author Contributions

All authors listed, have made substantial, direct and intellectual contribution to the work, and approved it for publication.

## Conflict of Interest Statement

The authors declare that the research was conducted in the absence of any commercial or financial relationships that could be construed as a potential conflict of interest. The handling Editor declared a shared affiliation, though no other collaboration, with the author AR and states that the process nevertheless met the standards of a fair and objective review.
